# The Making of a Good Carer: Dementia and Family Caregiving in an Era of Refamilization and Responsibilization in the Nordic Context

**DOI:** 10.1007/s10912-025-09944-7

**Published:** 2025-04-03

**Authors:** Åsa Alftberg

**Affiliations:** Department of Social Work, Faculty of Health and Society, Malmö University, SE–205 06 Malmö, Sweden

**Keywords:** Dementia, Informal caregiving, Family carers, Responsibilization, Textual analysis

## Abstract

A growing number of older people with dementia are continuing to live in their own homes for prolonged periods, leading to a growing number of family carers. An aging population and neoliberal austerity politics are contributing to increased family-provided care instead of formal care services. This is particularly noticeable in the Nordic context, where the welfare state has traditionally been based on universalizing policies designed to mitigate inequalities. The aim of this article is to explore societal expectations of family caregivers and the rhetoric surrounding family caregiving through analyzing a Swedish handbook entitled *Dementia for Family Carers: A Handbook from the Swedish Dementia Centre.* The textual analysis identifies the various responsibilities expected of family caregivers and illustrates how this responsibility can best be designed. The responsibility of family carers is perceived as natural and self-evident, especially in the context of spouses or partners. In such relationships, when caring for someone with dementia, the expectation is that the carer will transition into a caregiving role rather than continuing to be a life partner. Family caregivers are also expected to be central coordinators of formal and informal care. Furthermore, the responsibility includes the carers’ own self-care and ensuring they have the necessary knowledge and support. In tandem with the refamilization process, in which more family carers are providing care for relatives, idealizing and norm-making processes of family caregiving are emerging. This responsibilization process is crafting conceptions of the good carer, one who is responsible for relatives and formal care, while also prioritizing their own well-being.

## Introduction

In her article “The Gentleman Vanishes: Dementia, Caretaking and the Life of the Mind,” Michelle Taillon Taylor (2017) described her experiences during her father’s prolonged battle with dementia. As she navigated her role as a caregiver, her perception of her own agency underwent a transformation:

…the increasingly chaotic fallout resulting from my father’s illness gradually challenged my own naïve, wistful assumption: that I had agency in my world. […] But with the progression of my father’s illness, my mother and I repeatedly confronted muddy ethical dilemmas about his care, logistical problems defying resolution, and endless, ineffectual administrative tasks. I learned to recalibrate my personal and professional objectives repeatedly, to adjust to evolving circumstances. Flexibility, rather than rational planning, became my most effective survival strategy, and my sense of agency was replaced with a sense of contingency. (Taylor 2017, 53)

Taylor’s experiences resonate with the experiences of many family carers. In her text, Taylor (2017) posed an important question: How can we care for each other and still lead our own lives? In the Swedish context, this is of growing concern since reductions in eldercare services, combined with an aging population, have led to an increased need for family caregiving. This article explores societal expectations of family caregivers and the rhetoric surrounding family caregiving in order to understand the advanced neoliberal exercise of power and how it takes shape in and has consequences for family caregiving. This exploration is facilitated through the analysis of a handbook entitled *Dementia for Family Carers: A Handbook from the Swedish Dementia Centre* (Demenscentrum 2021). The handbook’s objective is to support and strengthen family caregivers in their challenging situations. It provides guidance on how family members and close friends should assist people living with dementia in their daily lives. In that way, the content of the handbook refers back to Taylor’s question and offers insights into society’s discourse on family caregiving in Sweden. From a medical humanities perspective, placing dementia and family caregiving in its cultural and social context can highlight norms and values that surround family caregiving in relation to dementia, and how those shape our understanding of the family carer. The handbook thus serves as an example of power executed from a position of societal expertise toward family carers who care for people living with dementia. Dementia is both a biomedical phenomenon (i.e., a diagnosis) and the lived experience of a fatal disease (for the affected individuals and their families), but it is also a social phenomenon. In the Swedish or Nordic context, it is important to further explore the meaning of family caregiving and the social construction of family carers in relation to, and as part of, the welfare state.

### Dementia and family caregiving

Dementia encompasses various diseases that affect cognitive abilities, such as memory function, as well as communicative, social, and emotional abilities by damaging and destroying parts of the brain, a process largely linked to aging (Hydén and Rahman 2021). It is considered a significant global healthcare challenge (Nguyen and Li 2020), and the prevalence of dementia is growing in tandem with the aging population worldwide (Odzakovic et al. 2019).

Cultural beliefs and norms about dementia often revolve around the fear of losing one’s self, as cognition and memory are considered two essential attributes of humanness and are equated with selfhood in Western culture (Alftberg and Rosenqvist 2017; Basting 2003). People living with dementia are often perceived as having lost their unique personality and identity, based on the notion that dementia dissolves the selfhood of the individual (Kontos 2004, 2006, 2012). Furthermore, the stigma attached to dementia affects both those living with dementia and their families (Nguyen and Li 2020).

A growing number of older people with dementia are continuing to live in their own homes for prolonged periods. Consequently, there is a growing number of family caregivers, also referred to as informal carers^1^ (Andréasson 2021; Wimo et al. 2018). Swedish caregivers mainly comprise older partners co-dwelling in a joint household and/or adult children, with a significant majority being women (Vicente et al. 2022). Replacing family caregivers with professional care providers would be costly at around SEK 193.6 billion per year (Ekman et al. 2021).

The care, support, and help they regularly provide to their relatives are deemed fundamental to the well-being of those living with dementia. Nevertheless, family caregivers, especially those providing more intensive care, are recognized as a vulnerable group facing increased risks of poor health, social isolation, financial strain, poverty, and reduced overall quality of life (Hanson 2023). Indeed, carers of people living with dementia are the subgroup most affected by depression, with one in three experiencing clinical depression (Huang 2022). They often report high levels of stress and feel pressured to make significant commitments to care for, help, and/or support their relatives with dementia (Johansson 2024; Lethin et al. 2019). In Sweden, the circumstances of family caregivers have even been characterized as a public health issue (National Board of Health and Welfare [Socialstyrelsen] 2020).

Family carers are often driven by a sense of moral duty and emotional affinity and sympathy, as well as by necessity (Meulen and Wright 2012). The caregiving role is perceived as simultaneously both self-imposed and enforced (Ulmanen 2015; Whitaker 2009). A mismatch in communication and expectations between professional caregivers and other family members can further increase the feeling of coercion and diminish the sense of autonomy and choice in the caregiving role (Laparidou et al. 2019). Likewise, expectations related to gender, socioeconomic status, and ethnicity play a significant role in caregiving dynamics (Andréasson 2021; Forssell and Torres 2012; Friedemann and Buckwalter 2014; Milligan and Morbey 2016; Vicente et al. 2022; Wallroth 2016). For instance, daughters from the manual-working class are much more likely to provide care for their aged parents and tend to engage in more demanding caregiving roles (Szebehely and Trydegård 2012; von Saenger et al. 2023).

An emphasis on concepts and norms concerning health, medicine, and care is distinctive of research within the medical humanities (also called critical medical humanities) (Whitehead and Woods 2016). When issues related to health and caregiving are situated in a wider cultural, political, and social context, societal structures can be brought to light and discussed (Viney et al. 2015). Critical medical humanities “engage[s] with other critical discourses” (Macnaughton 2017, 235) to address inequalities and power relations, for instance, refamilization and responsibilization, as will be discussed below.

### From societal obligations to individual responsibilities

In a Nordic context, the welfare state has traditionally been based on universalizing policies designed to mitigate gender and class inequalities. In every Nordic country, the legislation states that children and other family members are not obligated to care for adults; instead, social service or care acts mandate that local authorities must provide care to those who are officially determined to require such services (Szebehely and Meagher 2018). However, previous research has shown rising societal pressures for family carers to assume greater responsibility for older and disabled family members, in Sweden as well as in the Nordic countries as a whole (Olin and Dunér 2016; Szebehely and Meagher 2018; Ulmanen and Szebehely 2015; Wittberg et al. 2024). Leinonen (2011) linked this development to an aging population and strained state finances, resulting in decreased eldercare services such as homecare and specialized housing. Neoliberal austerity politics have further contributed to this shift, making people increasingly dependent on family-provided care instead of formal care services (McPherson and Oute 2021). As pointed out by Szebehely and Meagher (2018), the Swedish welfare model and eldercare show signs of de-universalization—for example, those with more resources may exit the system to purchase superior services on the market, and some or all service provision is passing to private providers. The consequences of declining eldercare coverage since the 1980s are most likely an increase in family caregiving (Szebehely and Meagher 2018).

The increasing reliance on families for care can be described in terms of refamilization: a greater dependence on the family due to reduced access to formal care and welfare (Ulmanen and Szebehely 2015). For instance, the number of Swedish people aged 75 years and over who received care from relatives rose from 40 percent in 1988–1989 to 65% in 2010 (Ulmanen 2015). Additionally, there has been a shift toward responsibilization: placing the onus of care that previously rested on the welfare state on individuals and families instead (Juhila et al. 2017). The responsibilization process treats individuals and families, rather than publicly funded services, as key resources (Teghtsoonian 2009). A Swedish example of this is that, while an older person living with dementia is still granted support from municipal eldercare services, the structure of this can vary based on the family situation and potential family caregivers (Takter 2017).

The concepts of refamilization and responsibilization are particularly obvious in relation to aging and old age. In Sweden, a key factor is the ongoing reduction in nursing home beds (Szebehely and Meagher 2018), highlighting a shift on the part of the National Board of Health and Welfare [Socialstyrelsen] (2019) toward a policy of person-centered care provided locally (in Swedish, “god och nära vård”). This approach especially emphasizes community-based care, essentially care at home, suggesting an increased reliance on family members for caregiving (Nilsson 2023). A growing proportion of older people in Sweden have extensive care needs and continue to live at home, yet homecare services have not kept pace with their needs (Szebehely and Meagher 2018). This has led to a decrease in the voluntary nature of family caregiving. The issue is not only the extent of care that family carers are willing and able to provide, but also whether they can provide care at all. Consequently, the question becomes not *if* family members are expected to provide care, but rather *how much*.

## Methods and theoretical framework

The empirical material for this article is the text *Dementia for Family Carers: A Handbook from the Swedish Dementia Centre*^2^ published by the Swedish Dementia Center in 2021. The Center is an important national organization and key resource in the field of dementia. It operates as a foundation, commissioned by the Ministry of Health and Social Affairs and the National Board of Health and Welfare to establish a national center of excellence in dementia care. The Center’s mission is to advance knowledge and enhance the care of persons with dementia, with the goal of fostering a more dementia-friendly society (Demenscentrum 2024). In this article, the information and recommendations of the authorities and experts from this organization are considered part of a discourse on family caregiving.

The handbook, spanning 124 pages, is available online and in print. It serves as a guide for those caring for a relative living with dementia, discussing implicit expectations and perceptions regarding the family caregiving role. The text can be understood as part of a discourse that highlights how family carers are constructed as a category, and how to be a “good carer.” The text is part of a larger context in which ideas about dementia and family caregiving are conveyed and reproduced. While the handbook targets family carers in general, it frequently cites instances involving couples, one of whom is affected by dementia. It has a clearly supportive tone, aiming to guide family caregivers in finding a balance between the needs of the person living with dementia and their own personal needs.

A close reading of a single text is a common method in discourse analysis, as exemplified by Isaksson (2011), and in the field of medical humanities and narratives in medicine (see, e.g., Bernhardsson 2024). Any text and/or practice cannot constitute itself as a fixed meaning outside a particular domain—that is to say, a discourse (Isaksson 2011; Laclau and Mouffe 1990). As such, even a single text reflects, or rather reproduces, the discourse of which it is part. Furthermore, a discourse establishes the framework within which a phenomenon can be understood (Laclau and Mouffe 1990). For example, a phenomenon such as family caregiving can be understood and interpreted in different ways, yet it is confined to the boundaries delineated by the prevailing discourse. The way something is articulated—whether through speech, writing, or action—within the context of a given discourse also reveals the conceptions that are taken for granted and appear natural and self-evident (Foucault 1981).

The analysis was conducted through careful and repeated reading of the handbook, initially searching for how the caregivers were addressed, with an openness to multiple interpretations but centered on the articulation of caregivers’ responsibilities. In practice, this meant that paragraphs and sentences that illustrated the caregiver’s responsibility were highlighted and compiled in a document. The next step was to reread and sort this material into different forms of responsibility, which identified four themes. Quotations that distinctly illustrated the content of each theme were selected, translated from Swedish to English, and then proofread by the author.

The emphasis on responsibility stems from a theoretical framework that connects individual responsibility with societal governance and self-discipline (Foucault 1990, 1995). Foucault posited that individuals are socialized into actions perceived as good for both the individual and society in what is essentially a form of self-discipline (Foucault 1990, 1995). This self-discipline extends beyond social institutions, embodying a process that is not confined by institutional boundaries (Bartky 1990). The “natural” responsibility attributed to family caregivers can be regarded as socialization into a caregiving role considered beneficial for both the individual and society. However, this analysis allows for the examination and discussion of what is perceived as natural and self-evident. Engaging a critical perspective on family caregiving is underlined by medical humanities’ focus on societal structures that influence health and care, along with widening the sites where caregiving takes place (Viney et al. 2015).

Viewed from the perspective of governmentality studies, responsibility as a theoretical concept is closely connected to autonomy and the ability to make choices that enhance one’s overall well-being, health, safety, and quality of life, as well as the welfare of one’s family members (Juhila et al. 2017). The concept of responsibilization structures specific forms of interventions, including various institutional and professional programs designed to strengthen individuals’ responsibilities for their own lives (Juhila et al. 2017). According to Rose and Miller (2010), professional expertise (i.e., the complex of actors, powers, institutions, and bodies of knowledge) has a central role in establishing the possibility and legitimacy of government and responsibilization. Experts, embodying neutrality, authority, and skill, have emerged as a significant solution for administering our lives according to conceptions of what is good, healthy, normal, and efficient (Rose and Miller 2010). Such governmental technologies, or *responsibility projects*, emphasize individual conduct, training, and education, shifting the focus away from structural exclusion and explanations (Ilcan 2009). They aim to mobilize certain groups and are designed by experts to stimulate the assumption of individual responsibility (Cossman 2013). The handbook aligns with such projects by aiming to educate family carers and train them to be, and think of themselves as, carers.

## Findings: Different forms of responsibility

The findings based on the Swedish Dementia Center’s 2021 handbook identify various responsibilities expected of family caregivers. They also illustrate how this responsibility can best be designed. In the presentation of the findings, the concept of responsibility will be explored through four different themes: natural responsibility, responsibility for the person with dementia, responsibility toward formal care, and responsibility for oneself.^3^ The most obvious responsibility is the natural one, implying the supposedly normal approach to being a family carer. Likewise, the responsibility for the person with dementia seems to be an obvious responsibility when being a family carer. Perhaps more surprisingly is how the responsibility toward formal care is articulated, together with the self-care aspect concerning responsibility for oneself.

### Natural responsibility

The handbook portrays the responsibility of family caregivers to provide care to a relative as a given. Formulations such as “It often feels natural to be there one hundred percent for one’s husband or wife” (19) and “Family carers are today responsible for a very large part of informal care. Many people want and think it is natural to stand up for their loved ones” (24) shape the expected norm for family carers. The handbook suggests that providing care is normal and natural, implying that full commitment is expected. Consequently, to hesitate to take that responsibility is implicitly unnatural. Although the book’s primary aim is to offer support to carers, with the intention of confirming and recognizing their efforts, it fails to address the voluntariness of family caregiving, leaving this significant consideration unexplored.

The handbook highlights the transformation in spousal roles, in which mutual relationships evolve and various tasks are taken on by the healthy spouse. It posits that embracing the caregiver role over the spousal role is something one is expected to undertake: “If you understand that you are not a couple in that way anymore, but that you are a relative who cares and supports. If you have accepted this, it may feel a little easier” (78). This shift may affect the legitimacy of certain feelings and actions, potentially leading to guilt or shame when they pertain more to being a spouse than a caregiver. The expected transformation of the relationship within a couple characterizes the content of the book and is transparent in the following themes.

### Responsibility for the relative with dementia

Regarding taking responsibility for the relative living with dementia, the book outlines how family carers should approach their responsibility toward a relative living with dementia. It emphasizes offering support, but in considerate and appropriate ways. The person living with dementia should continue to feel needed, so caregivers are advised to not to intervene too much:

Both family carers and everyone who in their work comes into contact with people living with dementia should strive to strengthen their self-esteem. Try to contribute to positive feelings and experiences and prevent negative ones. This is about taking care of what is healthy and stimulating what is still working. Everyone wants to feel important and needed. Therefore, it is important to support but not take over too much. (41)

Furthermore, the importance of caregivers in supporting individuals with dementia is emphasized. They play a crucial part in motivating, reminding, and encouraging the person, which is essential for well-being. To boost the relative’s self-esteem, caregivers can engage them in enjoyable and fun-filled activities. Caregivers can also preserve the identity and abilities of the person living with dementia: “In order to preserve the identity and abilities for as long as possible, the family carers should nurture and support these” (45). This is referred to as “functioning as an ‘auxiliary self’” (45), which in turn could be understood as an element of the transformation of the relationship between the parties involved. Thus, the expectations placed on caregivers are high and require a great level of commitment.

In situations in which family caregivers encounter, for example, violence from persons living with dementia, it is important to seek professional support. Additionally, caregivers should reflect on their own behavior to determine whether they may have inadvertently contributed to the violent situation:

It could even be about your loved one hitting you. But people do not hit someone out of nowhere. It could be that she was not suitably treated. Or she wants to express her frustration. Naturally, you as a carer should seek professional help when something like this happens. […] For the staff—and perhaps also for you as a family carer—it is still important to think about your own behavior. Perhaps you have asked questions that the person living with dementia cannot answer? Does she not feel validated and seen? (99)

The presence of illness seemingly causes a shift in perspective regarding responsibility for violent behavior, placing the onus on the person subjected to the violence.

The book also acknowledges the challenges caregivers face in consistently treating relatives living with dementia appropriately. It suggests strategies for meeting these challenges, such as walking away for a while and cultivating patience:

It is of course unreasonable that you as a family carer should always be able to say and do the “right” thing. It is human to not always be able to keep calm and kindly answer the same question time and time again. When you feel yourself getting angry—because you probably will—try to step away for a while. […] It is important to arm yourself with patience as much as possible. (40)

The handbook suggests that while it is natural for caregivers to experience difficult emotions, they should strive to manage them appropriately. By stepping away for a while, for example, when feelings such as anger become too intense, the caregiver takes responsibility for appropriate treatment of the person with dementia.

### Responsibility toward formal care

The book highlights the critical role of family caregivers in dementia care, emphasizing their partnership with formal care providers (e.g., healthcare and social care). It underscores the dual benefit of formal care support, aiding both the relative living with dementia and the caregiver. Indeed, while formal care primarily targets the person living with dementia, it can also be seen as a form of support for family caregivers: “Remember that you will have to remain up to the task for many years. If your wife is at daycare three days a week, you may be able to cope better. Don’t wait until you are too tired” (24).

When meeting representatives of formal care, family caregivers play distinctive roles, depending on the formal care professional with whom they interact. With care managers, they must communicate issues that the person living with dementia cannot articulate. When dealing with homecare services, they should share insights into the relative’s life and routines so that the homecare can be adapted. It is also important for caregivers to take the initiative and establish clear communication protocols with homecare staff. There may also be a variety of care contacts that the caregiver is expected to “keep track of and coordinate, as well as medications and sometimes also medical equipment. Of course, this may require patience and feel time-consuming. It is important to feel that you have control over all contacts” (32). Accordingly, family carers are expected to maintain a comprehensive understanding of the situation and effectively coordinate care.

The book also emphasizes the necessity of family caregivers being able to “let go” to prevent burnout, even in situations in which homecare services can be experienced as uncomfortable:

If you are a couple living together, it may feel uncomfortable to let strangers into the home. It can be difficult for you as a relative to let go and it can feel strange that your home has also become someone else’s workplace. But keep in mind that you also have to cope as a family carer, and no one is helped by you wearing yourself out and perhaps falling ill too. (32)

Family carers are expected to delegate certain care responsibilities to the homecare services when necessary. Family carers should not only provide care, but also recognize the appropriate time to allow formal care to assume responsibility. This balance is crucial for maintaining the family carers’ health and well-being, as will be further developed below.

### Responsibility for oneself

As demonstrated, the book emphasizes that family caregiving means having significant responsibility for the person living with dementia, in relation to both personal care and formal care. However, it also emphasizes the critical need for family carers to take care of themselves. Caregivers are expected to take responsibility for themselves by establishing limits to prevent burnout: “But remember that you have to try to put up boundaries for how much you want and can do as a family carer. No one benefits if you, as a carer, exhaust yourself” (19). The book reminds caregivers that not taking responsibility for oneself could have dire consequences for their loved ones: “Because what happens to your loved one if you wear yourself out and fall ill?” (24). Clearly, the book underscores that if the carers fall ill, the primary concern is the impact on the persons living with dementia. The role of caregiving seems to be so prominent that it tends to overshadow other aspects of the caregivers’ life, reducing their identity to that of carers, nothing more.

The book includes a chapter entitled “Don’t forget yourself” (66), which underscores the importance of self-care and not taking on too much:

As a carer, you usually come second, when you are expected to help. There are many positive sides to helping a loved one. But it is not selfish to also think about yourself. There are too many examples of family carers falling ill because they took on too much. […] Put your oxygen mask on first, before helping others. (66)

The metaphor of the oxygen mask, familiar from airplane safety communications, highlights the necessity of carers’ prioritizing their own self-care if they are to effectively take responsibility for their relatives. Family carers risk falling ill if they take on too much, highlighting the importance of self-care. Consequently, carers who overextend themselves and become ill may need to reflect on their self-care practices. This discussion does not address the structural and organizational conditions that affect the workload of family carers and whether they are able to “let go.” Such conditions include the shortcomings of homecare services, such as a lack of trained staff, and reduced access to nursing homes.

Another responsibility is to seek knowledge and support: “It is useful to seek support and help early […]. You also need to equip yourself with knowledge of what help is available and what rights you both have” (19). The aim of this support and knowledge is apparently to enable continuation of the caregiving role: “It is quite natural for carers to need support of various kinds to cope throughout the years with a person living with dementia” (66). The knowledge that carers are deemed to need primarily pertains to aspects of the disease, with the goal of simplifying life for both carers and their relatives. It also involves gaining “knowledge and advice on how to be able to live a continued good life” (21). Thus, the carer’s good life presupposes being knowledgeable and responsible, with the act of caring extending to both the person living with dementia and the carer themselves.

## Discussion

Focusing on dementia and family caregiving, a medical humanities perspective can reveal how care involves social, cultural, and political aspects, and ultimately says something about the human existential condition. The political setting of family caregiving in the Nordic context is that the Nordic welfare state has attempted to promote social cohesion and solidarity, and its societal safety nets and welfare systems are more extensive than in many other countries (Zechner et al. 2022). Nevertheless, neoliberal, marketized, and financialized care arrangements, based on self-caring citizens, are known to increase inequalities (Andersson and Kvist 2015; Hansen et al. 2021). Family caregiving illustrates the transfer of obligations from the welfare state back to individuals and families. In tandem with this refamilization process, in which more family carers are providing care for relatives, idealizing and norm-making processes of family caregiving has emerged. This responsibilization process encompasses not only increasing individual responsibility but also the various ways in which responsibility should be designed so that one can be a “good carer”.

The findings highlight the large amount and particular forms of responsibility that family carers are encouraged and even expected to take. A good carer makes sure to be well-informed and competent in all aspects related to the condition and health of the person living with dementia. Still, the responsibility concerns not only the ill relative, as self-care is also vital. Maintaining one’s health is crucial to ensure the ability to cope and provide care for as long as possible. The logic of responsibilization is that the caregivers, according to Palęcka (2023), should accept difficult circumstances and seek solutions in their behavior, thoughts, and emotions, which all can be managed with self-care tools. Thus, individualized self-care, i.e., in which people are increasingly expected to take responsibility for and promote their own health and take care of themselves, has become a technique of governing carers (Palęcka 2023).

The responsibility of family carers is perceived as natural and self-evident, especially in the context of spouses or partners. In such relationships, when caring for someone living with dementia, the expectation is that the carer will transition into a caregiving role rather than continuing to be a life partner. The responsibility can be described as an “auxiliary self”—that is, the carer becomes an extension of the person living with dementia, providing constant support and affirmation. Family caregivers must also engage in self-reflection and handle difficult emotions, for example, by demonstrating patience. However, such reasoning raises critical questions: What are the consequences of assuming a carer role instead of a partner role? How does this affect the perception of the person living with dementia? If the family carer is regarded as nothing more than a caregiver, there is a risk that the person living with dementia will be reduced to nothing more than a care recipient. This maintains the dehumanizing discourse on dementia, in which people living with dementia are presumed to lose personhood as well as become degendered (Sandberg 2018). In reality, family carers often struggle to uphold their relationship to the person living with dementia. Spouses may try to sustain subjectivity for those living with dementia through gendered recognition and by emphasizing their roles as husbands and wives (Sandberg 2021). The feeling of couplehood may be facilitated through diverse strategies, such as recalling mutual memories, viewing photos from the past, and creating brief moments of connectedness, reciprocity, and interdependence (Førsund et al. 2015). These aspects are downplayed in the book and the message is instead to give up on such a relationship and accept being a caregiver, an exchange of relationships that could be overwhelming. Nonetheless, McPherson and Oute (2021) showed how the responsibilization of caregivers paradoxically downplays, or entirely neglects, the perspectives and burdens of disrupted families and broken relationships. Difficult feelings such as grief and guilt, and losing a sense of agency, have no place in the rational choice-making concerning one’s life that is the core of responsibilization (Juhila et al. 2017). On the other hand, feelings of guilt and shame are an important way of governing carers: self-regulatory techniques “can be installed in citizens that will align their personal choices with the ends of government” (Rose and Miller 2010, 286). Central to the logic of responsibilization, supposedly supportive messages can easily shift into blame and guilt.

While the handbook from the Swedish Dementia Center serves a crucial purpose—to support carers in their challenging roles—it simultaneously legitimizes the ongoing shift from societal to familial caregiving. The book can be interpreted as part of a responsibility project. The instructions concerning family carers’ conduct and knowledge are very much in line with the idea of self-governed citizens made responsible for the welfare of family members (Cossman 2013; Juhila et al. 2017). It “produces new ways for certain individuals and groups to think about and act programmatically on ‘their’ problems and ‘their’ relations with others” (Ilcan 2009, 221). For family carers, this means thinking about and acting on problems typical of family caregivers and their relations with others, such as the person living with dementia, other family members, as well as professionals in social care and healthcare. In short, they are mobilized as carers: a particular form of responsible citizens. The gendered aspects of this should also be considered. Even in countries with developed public welfare services, women continue to bear the main responsibility for care (Ulmanen 2022). The care of older people has traditionally been described as a core area of the Nordic model (Andersson and Kvist 2015), but when care work in the public sector is reduced—eldercare being a prominent example of this in the Nordic context (Szebehely and Meagher 2018)—it increases family caregiving and female responsibility. The Nordic welfare states, characterized as women friendly (Hernes 1987), might be transforming in this regard through the processes of refamilization and responsibilization.

In the future, family caregiving is likely to become even more important due to an aging population and austerity measures (Zechner et al. 2022). When discussing family carers’ conditions, health risks, and need for support, societal expectations in the form of increasing individual responsibility and normative beliefs concerning how to be a good carer must be taken into account. Otherwise, we will not be able to fully understand, or ease, the situation of family carers.

## Endnotes

^1 ^I use the term “family caregivers” rather than “informal carers” to emphasize the pre-existing primary relationship that caregiving augments. I also regard the concept of family as a rather broad category that encompasses close relationships beyond just kinship.

^2 ^My translation; the Swedish title is *Om demenssjukdom för anhöriga: En handbok från Svenskt Demenscentrum*. The book from 2021 is an updated second edition of an earlier version from 2014.

^3 ^The following quotations and descriptions from the book have previously been published in a chapter in Swedish (Alftberg 2022).
